# Preclinical evaluation of the Versius surgical system: A next‐generation surgical robot for use in minimal access prostate surgery

**DOI:** 10.1002/bco2.233

**Published:** 2023-03-13

**Authors:** Nikhil Vasdev, Philip Charlesworth, Mark Slack, Jim Adshead

**Affiliations:** ^1^ Hertfordshire and Bedfordshire Urological Cancer Centre Lister Hospital Stevenage UK; ^2^ School of Life and Medical Sciences University of Hertfordshire Hertfordshire UK; ^3^ Royal Berkshire Hospital Reading UK; ^4^ CMR Surgical Cambridge UK

**Keywords:** cystectomy, minimal access surgery, prostatectomy, robot‐assisted surgery, urological surgical procedures

## Abstract

**Objectives:**

To evaluate the Versius surgical system for robot‐assisted prostatectomy in a preclinical cadaveric model using varying system setups and collect surgeon feedback on the performance of the system and instruments, in line with IDEAL‐D recommendations.

**Materials and methods:**

Procedures were performed in cadaveric specimens by consultant urological surgeons to evaluate system performance in completing the surgical steps required for a prostatectomy. Procedures were conducted using either a 3‐arm or 4‐arm bedside unit (BSU) setup. Optimal port placements and BSU layouts were determined and surgeon feedback collected. Procedure success was defined as the satisfactory completion of all steps of the procedure, according to the operating surgeon.

**Results:**

All four prostatectomies were successfully completed; two were completed with a 3‐arm BSU setup and two using a 4‐arm BSU setup. Small adjustments were made to the port and BSU positioning, according to surgeon preference, in order to complete the surgical steps. The surgeons noted some instrument difficulties with the Monopolar Curved Scissor tip and the Needle Holders, which were subsequently refined between the first and second sessions of the study, in line with surgeon feedback. Three cystectomies were also successfully completed, demonstrating the capability of the system to perform additional urological procedures.

**Conclusions:**

This study provides a preclinical assessment of a next‐generation surgical robot for prostatectomies. All procedures were completed successfully, and port and BSU positions were validated, thus supporting the progression of the system to further clinical development according to the IDEAL‐D framework.

## 1 INTRODUCTION

Minimal access surgery (MAS) has been utilised in urological surgeries for several decades and is well established in prostate procedures.^1^, ^2^ Prostatectomy by MAS offers significant benefits over open surgery with shorter catheterisation time, less blood loss, less post‐operative pain, shorter hospital stay and recovery, lower rates of complications and comparable oncological outcomes.^3^, ^4^ However, MAS is associated with certain limitations, such as a lack of ergonomically designed surgical instruments, restricted reach and access of instruments, limited haptic feedback and the lack of depth perception resulting from two‐dimensional visualisation.^5^ Ultimately, such limitations can lead to an extensive learning curve for surgeons.^6^, ^7^ Additionally, during MAS procedures, surgeons often experience muscular strains and fatigue resulting from asymmetric static positioning and hunched postures.^5^, ^8^ These physical burdens are exacerbated by the technically challenging nature of urological procedures, where the surgeon must operate in a parallel axis to the pelvis.^5^

Robotic assistance in MAS may overcome some of the challenges associated with conventional MAS, retaining the advantages of a minimally invasive approach but often with greater technical ease and a shallower learning curve.^9^ Robotic systems can offer an enhanced three‐dimensional view, increased magnification of the surgical field, improved manual dexterity within the confines of the pelvis, tremor filtration and improved ergonomics.^6^ These advantages can reduce the learning curve associated with MAS, thus increasing the accessibility of MAS and enabling surgeons to perform more complex urological procedures.^10^, ^11^ Moreover, implementation of robotic systems in urological procedures could improve the management of prostate cancer, enabling the development of several techniques (including nerve‐sparing techniques) that improve functional and oncological outcomes.^12^, ^13^ Despite these significant advantages, there is scope for improvement in robotic systems, on account of the increased use of operating room (OR) space, possibility of equipment malfunction, initial learning curve, training of medical personnel and high costs of purchase and maintenance.^14^, ^15^

The Versius surgical system (CMR Surgical, Cambridge, UK) is a tele‐operated robotic surgical system developed for use in MAS.^16^ The device was developed with the aim of improving surgical outcomes for patients and to better meet the needs of surgeons; its design was refined iteratively according to end‐user feedback from surgeons.^17^

The system comprises a surgeon console with hand controllers and a head‐up display (HUD), a visualisation bedside unit (BSU) with an endoscopic camera, and up to four instrument BSUs. The HUD provides the surgeon with a three‐dimensional, high‐definition visual from the endoscopic camera.^16^, ^17^ The open console design of the device enables ease of communication between surgeons and their teams throughout surgical procedures, while also providing flexibility with a seated or standing operating position. The device is operated by hand controllers, which are ergonomically designed in the style of a ‘game controller’ and can accommodate a range of hand sizes.^16^, ^17^ The device instruments mimic the articulation of the human arm, which, together with the wristed joint of the instruments, provides seven degrees of freedom at the instrument tip, enabling greater surgical access compared with conventional MAS.^16^, ^17^ Development of a fenestrated bipolar device and an energy sealer device for this robotic system is currently ongoing. Additionally, the compact and mobile BSUs allow the system to be used within standard ORs and easily moved between ORs for maximum flexibility.^16^, ^17^

The IDEAL‐D (Idea, Development, Exploration, Assessment, Long‐term study–Devices) framework provides recommendations for generating a detailed evidence base throughout the medical device development process.^18^, ^19^, ^20^ Previous studies have provided evidence of the development and operational safety of the system, according to Stage 0.^16^, ^17^ Preclinical studies have provided proof of concept for the use of the device in a range of procedures for gynaecology, renal and urology, and general and colorectal surgery (Stage 1).^21^, ^22^, ^23^, ^24^ These studies have supported continuation to in‐human clinical trials of the surgical robot in hysterectomy and cholecystectomy surgeries,^25^, ^26^ and implementation in other surgical specialties is ongoing (Stage 2).

The preclinical study described herein aimed to evaluate the suitability of the device for use in prostatectomies, in line with the IDEAL‐D framework (Stage 1). The primary objective was to evaluate the use of the system in completing the surgical steps required for a prostatectomy using either a 3‐arm or 4‐arm BSU setup in a preclinical cadaveric model. Secondary objectives were to determine the optimum port placements and BSU layouts for a prostatectomy using either a 3‐arm or 4‐arm BSU setup and collect surgeon feedback on the performance of the system and instruments.

## 2 MATERIALS AND METHODS

All cadaver procedures were conducted at The Evelyn Cambridge Surgical Training Centre, Cambridge, UK. All cadavers were donated with consent.

The first session of the study was conducted in July 2020 and included the 3‐arm setup procedures, and the 4‐arm setup procedures were performed in a second session in December 2020. The initial 3‐arm approach was used to optimise port placement. The room setup was designed to mimic that of a real clinical OR, including a full‐length operating table to provide realistic spatial constraints for the setup of the system and the permitted use of additional laparoscopic equipment to help complete the procedure: Monopolar scissors, bipolar Maryland instruments and blunt graspers were used during the procedures.

Procedures were performed by a lead surgeon supported by surgical assistants. The lead surgeon performed the surgical steps of the procedure and evaluated the system in line with the objectives of the study. The surgical assistants carried out any additional manual tasks as instructed by the lead surgeon. Additional personnel present recorded port and BSU placements along with outcomes.

The three lead surgeons who participated in the study were practising, accredited, high‐volume consultant urological surgeons, as defined by >50 cases per annum for the procedures performed. The surgeons spent approximately 1 h on a training simulator developed specifically for the device, to refamiliarise themselves with the operating system and console, having completed the fully validated training program.^27^

**FIGURE 1:**
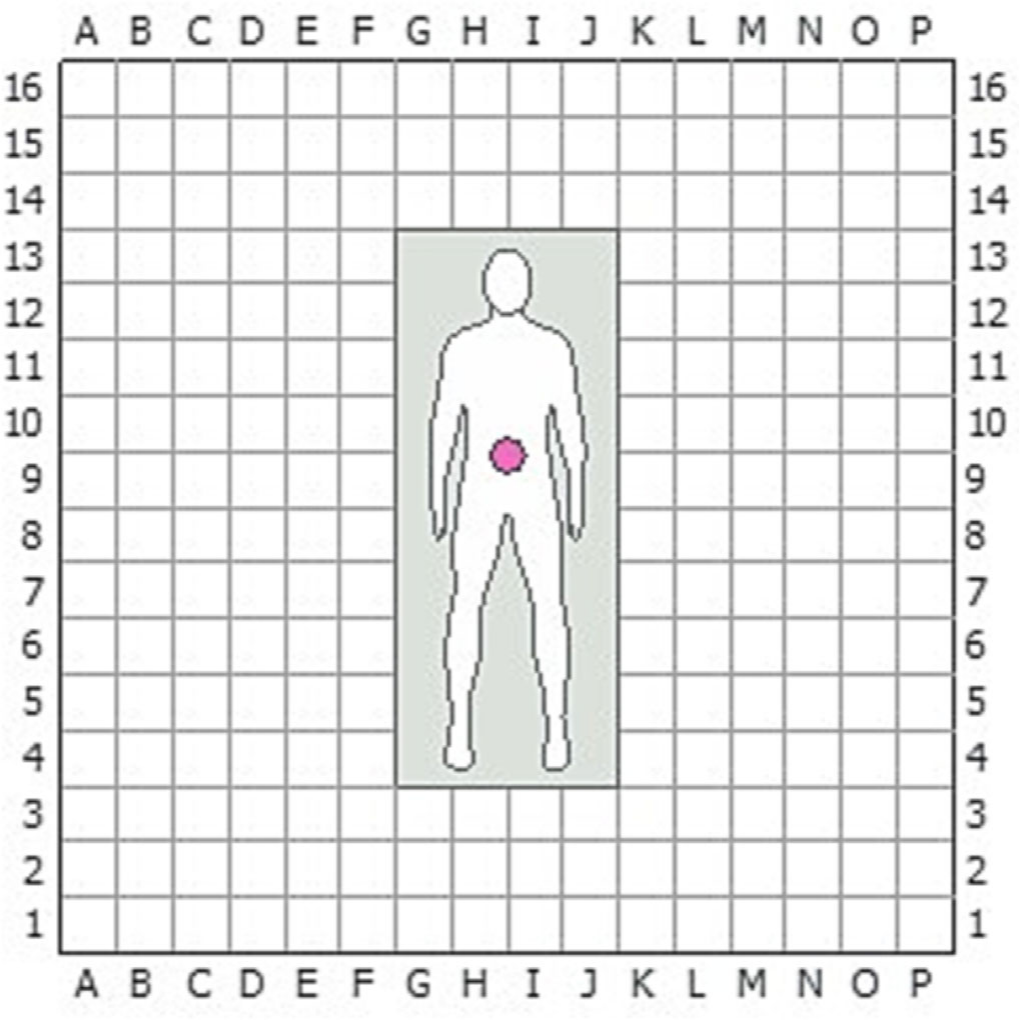
Grid used to record port and BSU positions. A grid of 20 cm × 20 cm squares was laid out on the OR floor (overall grid was 320 cm × 320 cm) to ensure standardised and reliable reporting of measurements. Pink circle indicates the umbilicus (where the midline crosses the supine‐umbilical line). BSU, bedside Unit; OR, operating room.

Prostatectomies were performed in four cadaveric specimens (torso to mid‐femur), who had not undergone previous prostate surgery, using a 3‐arm and a 4‐arm BSU setup (Online Resource 1). The port and BSU positions were determined based on the experience of the lead surgeon in performing the same procedure by conventional means. Port and BSU placements were recorded using a 320 cm × 320 cm grid laid out on the operating theatre floor (Figure 1). BSU positions were also recorded in relation to anatomical landmarks on the cadaver. Suitable positioning was defined by clear surgical access without arm clashing and a minimal need to reposition the BSUs. The precise surgical steps conducted, as well as instruments used, endoscope angles and electrosurgical settings, were recorded to confirm the completion of the prostatectomies. Procedure success was assessed by the ability of the system to perform each step of the procedure satisfactorily, as determined by the lead surgeon.

## 3 RESULTS

The cadavers represented body mass indices (BMIs) ranging from 24.3 to 32.0 kg/m^2^ (mean: 27.0 kg/m^2^). Four prostatectomies were performed, the surgical steps of which are outlined in Online Resource 2. Three cystectomies were also performed to demonstrate the ability of the system to perform additional urological procedures; the surgical steps for these procedures are outlined in Online Resource 3. All eight procedures were successfully completed (Table 1). Of these, two prostatectomies and one cystectomy were performed with a 3‐arm BSU setup. The other two prostatectomies and two cystectomies were performed using a 4‐arm BSU setup.

**TABLE 1:** Summary of procedures performed using either a 3‐arm or 4‐arm BSU setup and successful completion.

Procedure	Number performed	Number successfully completed
Procedures with 3‐arm BSU Setup		
Prostatectomy	2	2
Cystectomy	1	1
Procedures with 4‐arm BSU Setup		
Prostatectomy	2	2
Cystectomy	2	2

Abbreviation: BSU, bedside unit.

During the prostatectomies performed using a 3‐arm BSU setup, the port positioning was effective and all surgical steps were completed without any adjustment. The BSU positioning required minor adjustments. In one procedure, the visualisation BSU was exchanged with a replacement to avoid delays following a system alarm (caused by high pressure levels between the endoscope and operating trocar).

Throughout the prostatectomies performed using a 4‐arm BSU setup the port positioning required no adjustments to complete the surgical steps; however, the initial port placement was further lateral in the second procedure to compensate for the increased BMI. In the early stages of the first procedure, the positioning of the BSU was adjusted following instrument arm clashes that generated two medium‐priority alarms. Later in this procedure, the BSU setup was changed from two on the left and two on the right to three on the left and one on the right side, in order to allow greater access for the surgical assistant (this placement was also carried over into the second procedure). Minor adjustments to BSU positioning were needed during the second 4‐arm setup prostatectomy, once the BSUs were brought closer to the operating table to accommodate the larger BMI.

**FIGURE 2:**
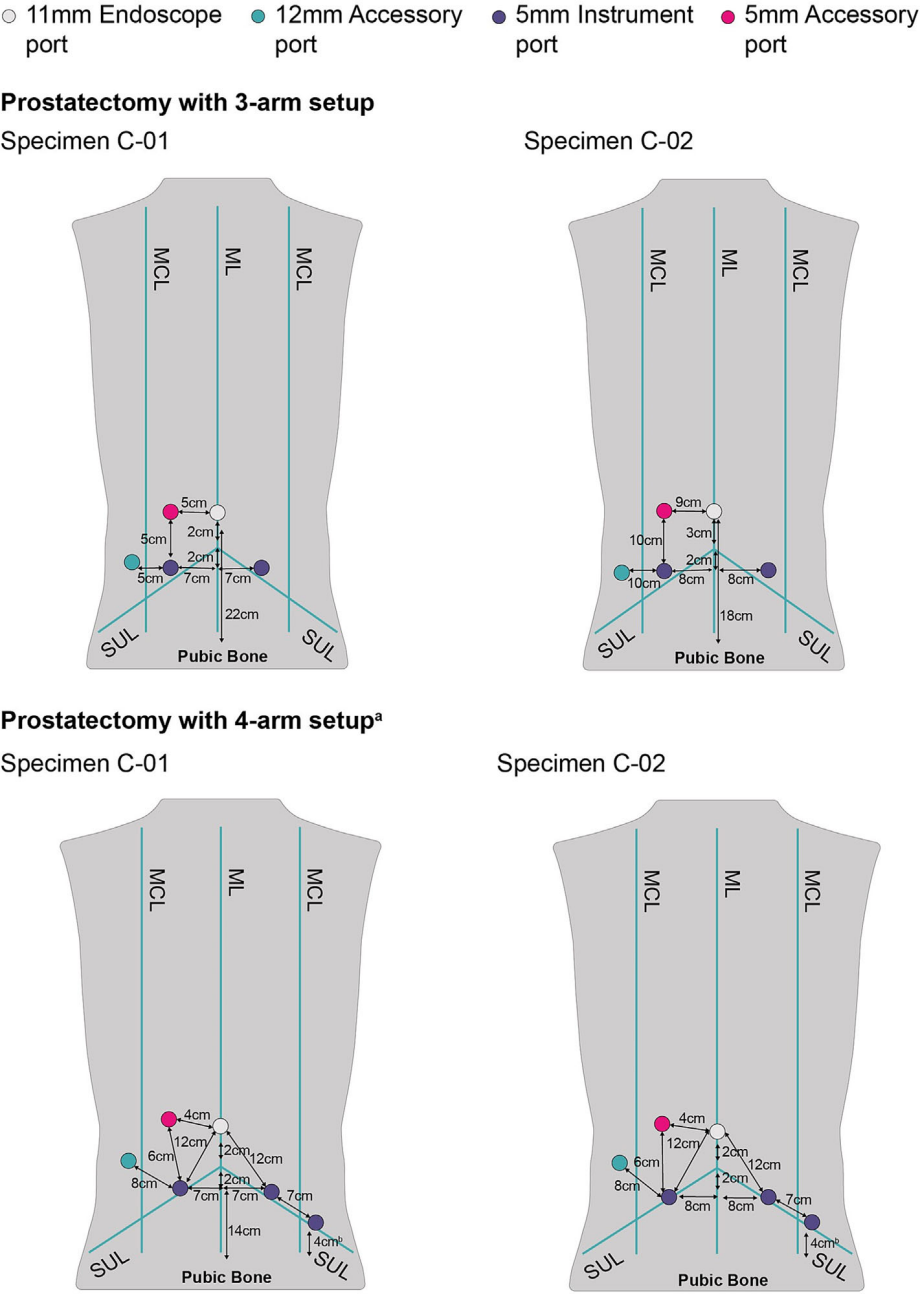
Port placements for prostatectomy using either a 3‐arm or 4‐arm BSU setup. ^a^Abdomen insufflated to 12 mmHg. ^b^4 cm from anterior superior iliac spine. BSU, bedside unit; ML, midline; MCL, midclavicular line; SUL, supine umbilical line.

**FIGURE 3:**
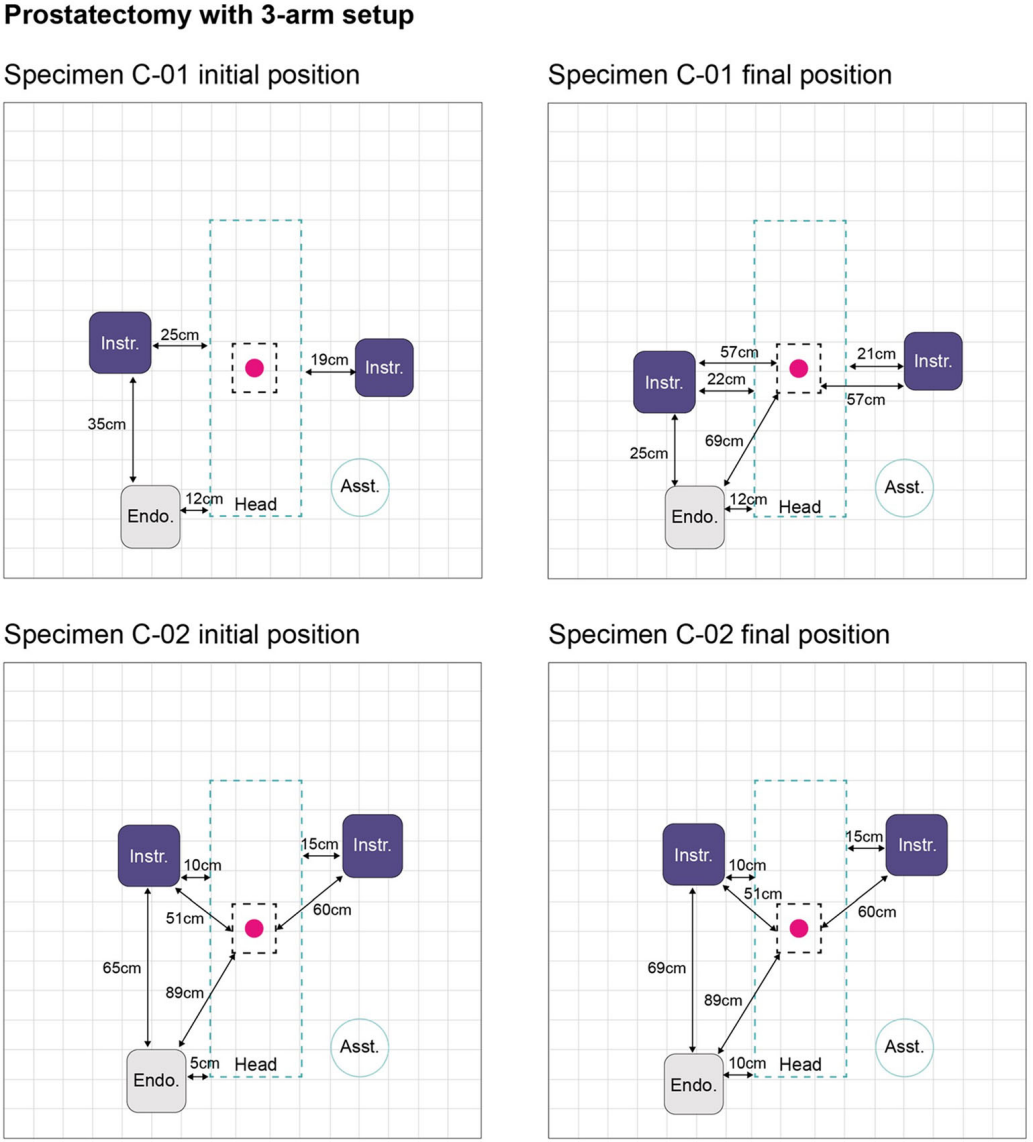
Initial and final BSU positions for prostatectomy using a 3‐arm BSU setup. Pink circle indicates the umbilicus (where the midline crosses the supine‐umbilical line). Specimen C‐01 table height at its lowest was 60 cm and at its highest was 90 cm. Specimen C‐02 table height at its lowest was 45 cm and at its highest was 91 cm. BSU, bedside unit; Asst., assistant; Endo., endoscope; Instr., instrument.

**FIGURE 4:**
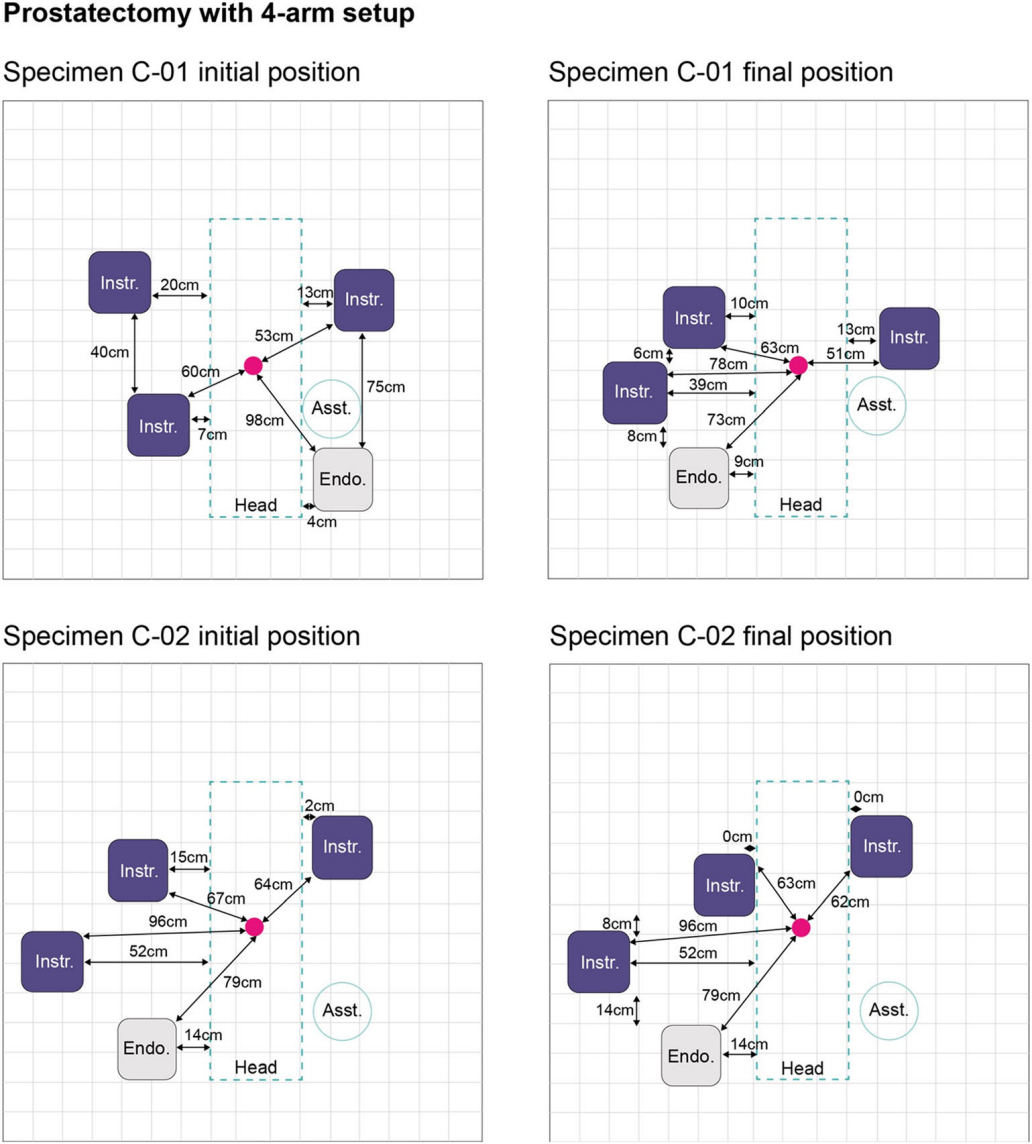
Initial and final BSU positions for prostatectomy using a 4‐arm BSU setup. Pink circle indicates the umbilicus (where the midline crosses the supine‐umbilical line). Specimen C‐01 table height at its lowest was 43 cm and at its highest was 102 cm. Specimen C‐02 table height at its lowest was 47 cm and at its highest was 96 cm. BSU, bedside unit; Asst., assistant; Endo., endoscope; Instr., instrument.

Figure 2 illustrates the port positioning configurations used by the surgeons for the 3‐arm and the 4‐arm BSU setups. All active instrument ports were placed below the umbilicus with the fourth instrument port placed 4 cm from the anterior superior iliac spine. The initial and final BSU positioning used by the surgeons for the 3‐arm and 4‐arm BSU setups are shown in Figures 3 and 4, respectively.

**FIGURE 5:**
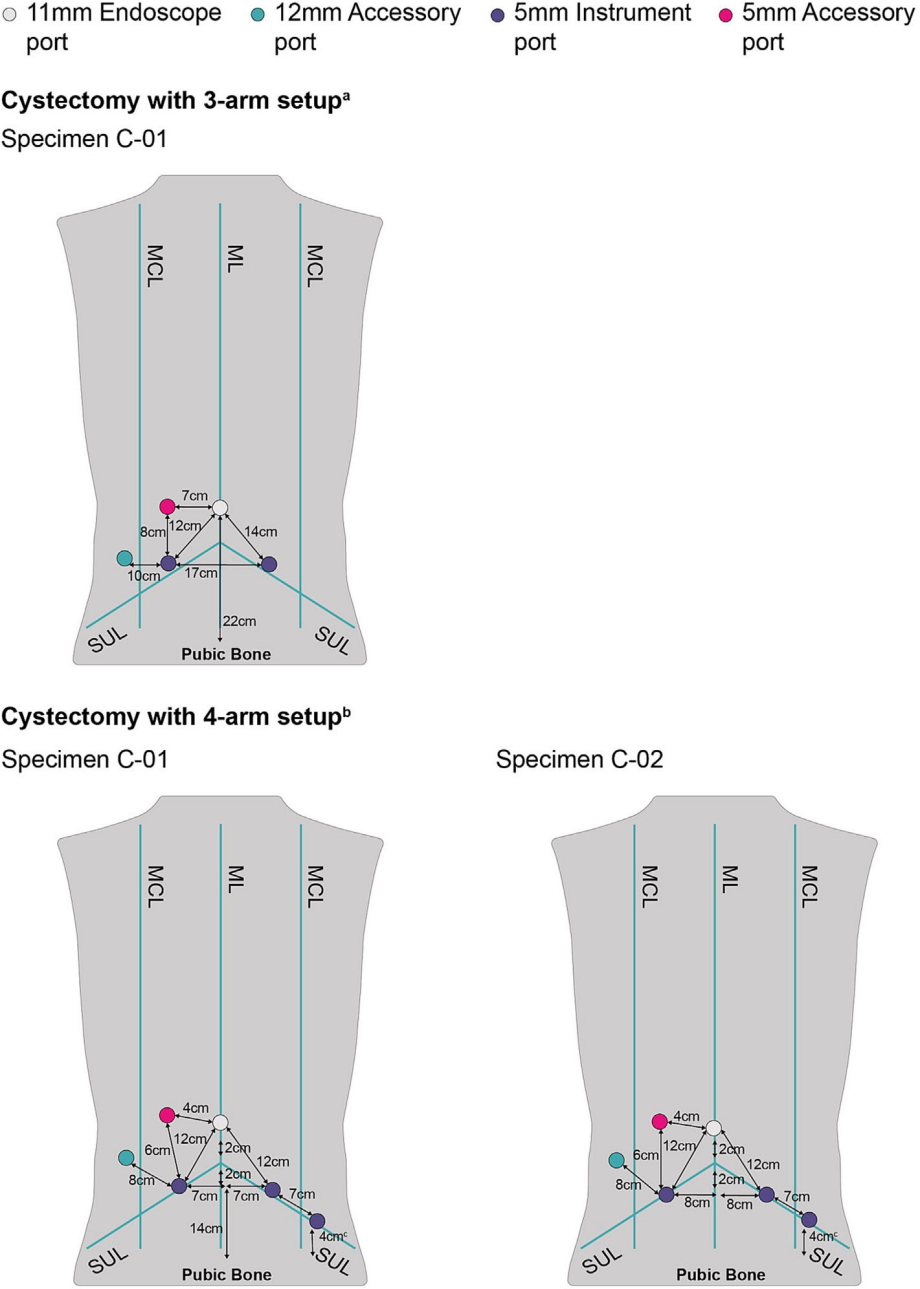
Port placements for cystectomy using either a 3‐arm or 4‐arm BSU setup. ^a^Instrument ports placed once insufflation was completed. ^b^Abdomen insufflated to 12 mmHg. ^c^4 cm from anterior superior iliac spine. BSU, bedside unit; ML, midline; MCL, midclavicular line; SUL, supine umbilical line.

**FIGURE 6:**
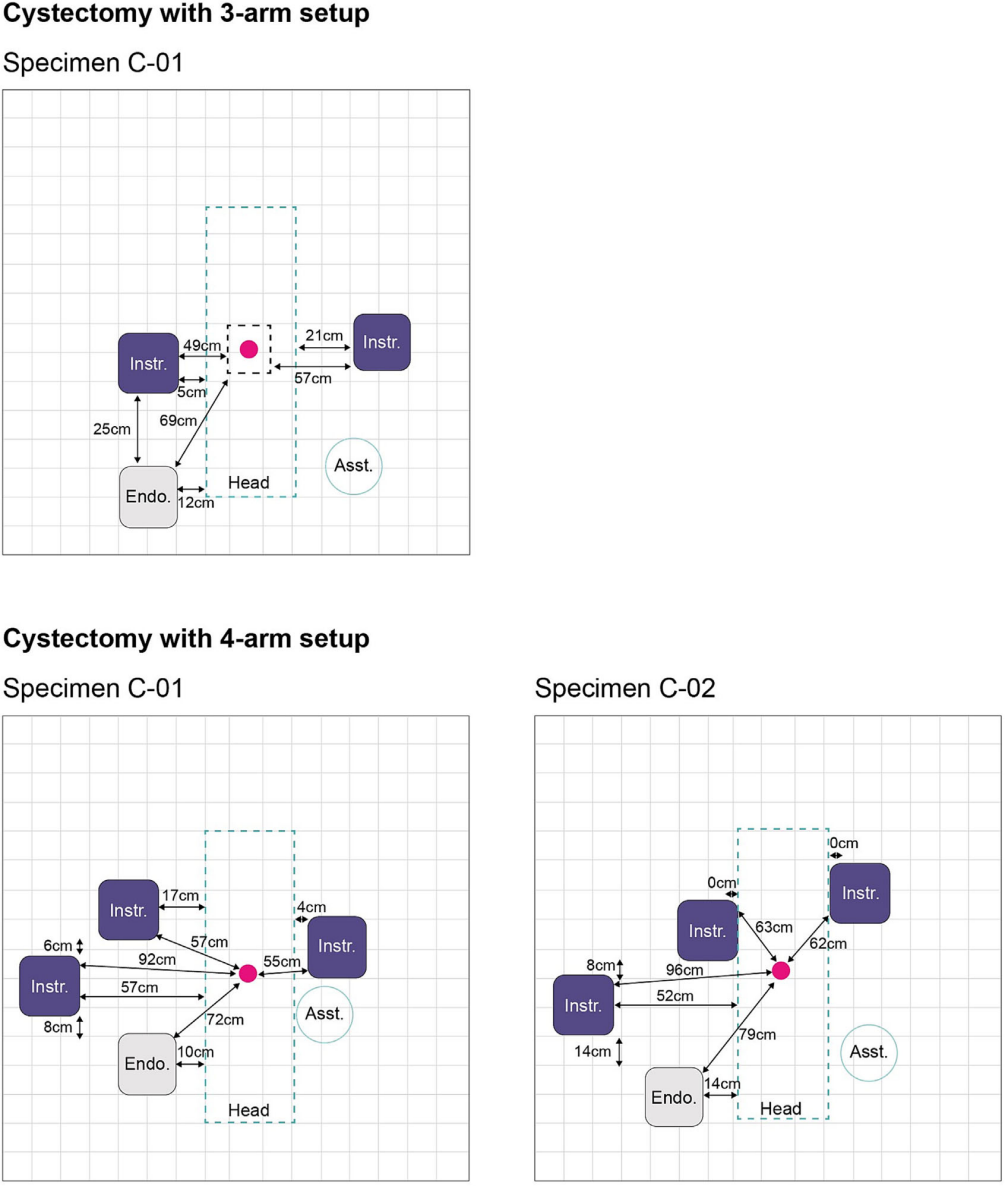
BSU positions for cystectomy using either a 3‐arm or 4‐arm BSU setup. Pink circle indicates the umbilicus (where the midline crosses the supine‐umbilical line). 3‐arm setup: specimen C‐01 table height at its lowest was 60 cm and at its highest was 90 cm. 4‐arm setup: specimen C‐01 table height at its lowest was 43 cm and at its highest was 102 cm; specimen C‐02 table height at its lowest was 47 cm and at its highest was 96 cm. BSU, bedside unit; Asst., assistant; Endo., endoscope; Instr., instrument.

The port positioning configurations and BSU positioning used by the surgeons during the cystectomy procedures for the 3‐arm and 4‐arm BSU setups are shown in Figures 5 and 6, respectively.

Having successfully met the primary objective, surgeon feedback was also collected as part of the secondary objectives. One of the lead surgeons observed that using the system with the 4‐arm setup had contributed to a reduction in operating time, had enabled better retraction and provided more accuracy when operating between the tissue planes. During one of the prostatectomies in the first session, the lead surgeon believed that a nerve‐sparing procedure could not be performed because of several instances of the Monopolar Curved Scissor tip yawing when cutting. The Needle Holders were also noted as causing difficulty in the first session when orienting the needle ahead of suturing and presented challenges with manoeuvrability. In both the first and second sessions, the surgeons suggested that a finer jaw variant of the Needle Holders would be superior and would enhance the intricacy of suturing.

Both the Monopolar Curved Scissors and the Needle Holders were refined between the sessions of the study in line with the surgeons' feedback, with surgeons noting the improvements in the scissors in the later session. A marked improvement to the Needle Holders was also noted by one of the surgeons.

## 4 DISCUSSION

The previous preclinical urological study in a cadaveric model was primarily focused on providing proof of concept of the device for robot‐assisted surgery in urological procedures.^23^ The focus of this study was to build upon existing preclinical knowledge and determine an effective and replicable 3‐arm and 4‐arm setup for both operating ports and BSUs in robot‐assisted prostatectomies.

The flexibility of the system and portability of the BSUs enabled adequate surgical access and reach in order to perform the procedures, including in specimens with high BMIs. The lead surgeons agreed that the system had assisted in successfully completing the surgical steps for prostatectomies with both the 3‐arm and 4‐arm BSU setup. The port placements and BSU positions were guided by findings from previous prostatectomy cadaveric studies.^23^ The placement of ports remained largely unchanged throughout the procedures, with only slight alterations to accommodate the increased BMI of one of the specimens. BSU positioning also underwent only minor adjustments once a suitable placement had been established and allowed adequate range of motion for each of the arms and instruments being used.

The design of this study was such that the 3‐arm BSU setup procedures were performed several months before the 4‐arm setup procedures, allowing for refinements to be made between the two sessions based on surgeon feedback; this included refinements to the Monopolar Curved Scissors and the Needle Holders. Refinements to the jaw style of the Needle Holder could be addressed prior to clinical studies.

Although this study aimed to mimic a real clinical setting as far as possible, a preclinical simulated setting cannot fully replicate a real‐world surgical environment. There are accepted physiological differences between cadavers and live human bodies, including the rigidity and discolouration of cadaveric tissue, poor handling fidelity and lack of bleeding. Surgeon performance may be influenced when using a novel operating system, because of the unfamiliarity of the preclinical operating environment and the device. Further, the pass/fail criteria for the successful completion of surgical procedures, although predetermined, were subjective and based only on the lead surgeons' feedback in relation to a live surgery. This binary measure of procedure success means that granular evaluation of more complex and nuanced individual surgical steps is difficult to capture.

## 5 CONCLUSION

This study provides a preclinical assessment of the robotic system for prostatectomy surgery in cadaveric models. Several types of urological procedures were tested, and port and BSU positions were validated in each of these; all surgeries were completed successfully. Overall, this preclinical study supports the progression of the device to further stages of development in clinical studies of prostate surgery, in broad alignment with the IDEAL‐D framework.

## AUTHOR CONTRIBUTIONS

Substantial contributions to study conception and design: Nikhil Vasdev, Philip Charlesworth, Mark Slack and Jim Adshead; substantial contributions to analysis and interpretation of the data: Nikhil Vasdev, Philip Charlesworth, Mark Slack and Jim Adshead; drafting the article or revising it critically for important intellectual content: Nikhil Vasdev, Philip Charlesworth, Mark Slack and Jim Adshead; final approval of the version of the article to be published: Nikhil Vasdev, Philip Charlesworth, Mark Slack and Jim Adshead.

## CONFLICT OF INTEREST STATEMENT

Nikhil Vasdev: No conflicts of interest or financial ties to disclose. Philip Charlesworth: No conflicts of interest or financial ties to disclose. Mark Slack: Chief Medical Officer and Co‐founder of CMR Surgical. Jim Adshead: Member of the advisory board for Lightpoint Medical.

## Supporting information


**Data S1.** Supporting Information
