# Supplementary material for: Preclinical evaluation of the Versius surgical system: A next‐generation surgical robot for use in minimal access prostate surgery
Source: BJUI Compass. 2023 Mar 13;4(4):482–90. doi: 10.1002/bco2.233 (PMC10268579; doi:10.1002/bco2.233)
Supplement: Supplementary file 1 — Data S1. Supporting Information [file BCO2-4-482-s001.docx]

**Article Title: Preclinical Evaluation of the Versius Surgical System: A Next-Generation Surgical Robot for Use in Minimal Access Prostate Surgery**

**Journal Name: BJUI Compass**

**Author Names: Nikhil Vasdev, Philip Charlesworth, Mark Slack, Jim Adshead**

**Corresponding Author: Dr Mark Slack (mark.slack@cmrsurgical.com; CMR Surgical Ltd, 1 Evolution Business Park, Milton Road, Cambridge CB24 9NG**

**SUPPLEMENTARY DATA**

**Online Resource 1.** Prostatectomy using a 3-arm and 4-arm BSU setup

BSU: bedside unit.


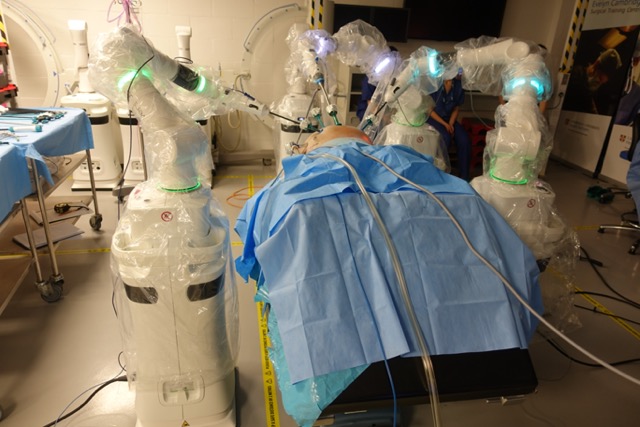

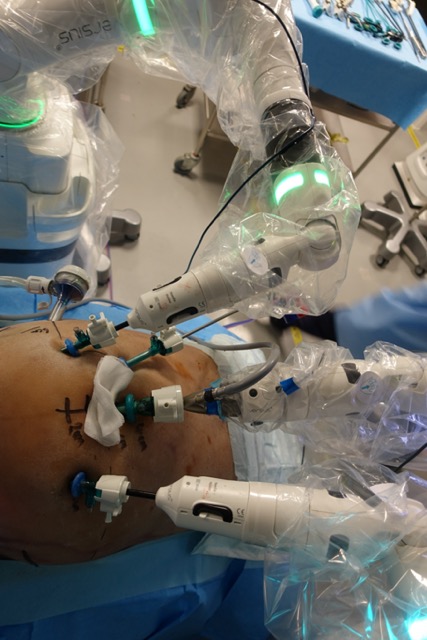

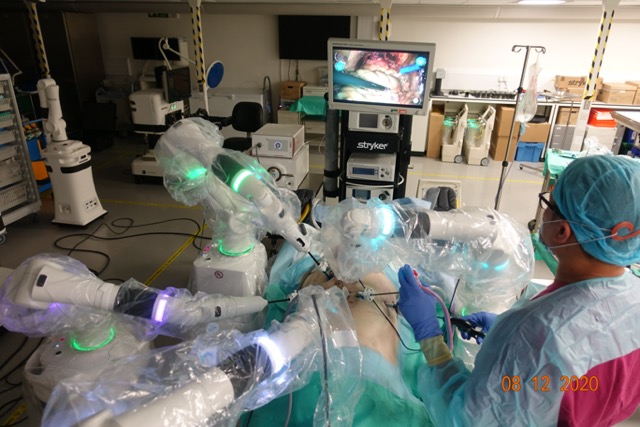

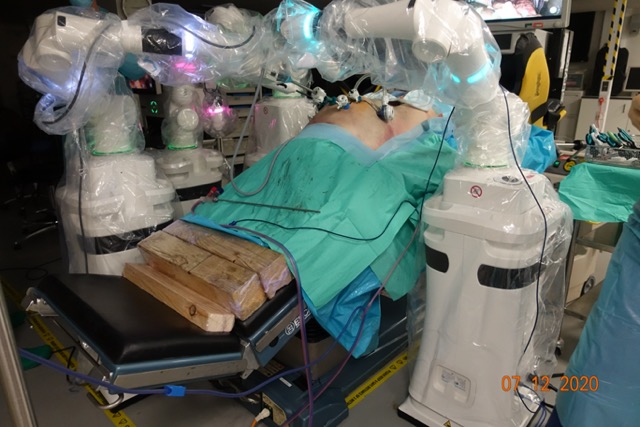


**Online Resource 2.** Surgical steps performed for prostatectomy with either a 3-arm or 4-arm BSU setup. The initial 3-arm approach was used to optimise port placement, with a 4-arm technique developed subsequently. It is the authors’ recommendation to adopt a 4-arm approach owing to additional tissue traction.

| **Prostatectomy Surgical Steps – 3-Arm BSU Setup** |
| --- |
| 1. Divide urachus and ligaments on side |
| 1. Expose fat on prostate |
| 1. Bladder neck dissection |
| 1. Open the bladder |
| 1. Divide SV and vas deferens |
| 1. Attempt nerve spare (optional) |
| 1. Rectal dissection |
| 1. Suture the DVC |
| 1. Prostate placed in retrieval bag |
| 1. Anastomosis |
| 1. Leak test |
| **Prostatectomy Surgical Steps – 4-Arm BSU Setup** |
| 1. Incise the peritoneum lateral to medial and lumbar ligaments from   the urachus to internal vein (right side) |
| 1. Drop the bladder (reaching further into pelvis) going into pubic arch |
| 1. Clean endopelvic fascia |
| 1. Remove fat from bladder |
| 1. Open endopelvic fascia and release endopelvic fascia |
| 1. Division of puboprostatic ligament |
| 1. Identify prostate-urethral junction |
| 1. Ligate DVC |
| 1. Incise anterior bladder neck |
| 1. Advance the catheter |
| 1. Incise posterior bladder neck |
| 1. Mobilise vas deferens and SV and then cut vas deferens and SV |
| 1. Mobilise Denonvilliers fascia to apex |
| 1. Divide pedicles |
| 1. Divide DVC |
| 1. Incise anterior urethra and deliver catheter |
| 1. Incise posterior urethra and deliver catheter |
| 1. Urethrovesical anastomosis |

BSU: bedside unit; DVC: dorsal venous complex; SV: seminal vesicle.

**Online Resource 3.** Surgical steps performed for cystectomy with either a 3-arm or 4-arm BSU setup. The initial 3-arm approach was used to optimise port placement, with a 4-arm technique developed subsequently. It is the authors’ recommendation to adopt a 4-arm approach owing to additional tissue traction.

| **Cystectomy Surgical Steps – 3-Arm BSU Setup** |
| --- |
| 1. Identify gonadal vein, psoas muscle and left ureter |
| 1. Expose and divide left VAS |
| 1. Expose endopelvic fissure on the left side |
| 1. Clip and divide left superior vesicle |
| 1. Clip and divide left ureter |
| 1. Clip and divide left superior vesicle pedicle |
| 1. Clip and divide right superior vesicle pedicle |
| 1. Expose and divide right VAS |
| 1. Identify right ureter |
| 1. Clip and divide median umbilical ligament |
| 1. Identify side plane of bladder and sigmoid colon |
| 1. Clip and divide right superior vesicle |
| 1. Clip and divide right ureter |
| 1. Complete right-side dissection to free the bladder |
| 1. Bladder placed in retrieval bag for extraction |
|  |
| **Cystectomy Surgical Steps – 4-Arm BSU Setup** |
| 1. Mobilise left ureter |
| 1. Tunnel under sigmoid to expose right ureter |
| 1. Dissect tissue surrounding right ureter |
| 1. Release sigmoid mesentery |
| 1. Release peritoneum on left and right side of bladder |
| 1. Clip and cut left ureter at level of superior vesical |
| 1. Pass left ureter through behind the sigmoid |
| 1. Perform left-sided lymph node dissection (level 3) |
| 1. Clip and cut right ureter |
| 1. Clip and cut the superior vesical artery |
| 1. Dissect the bladder off the back of the rectum |
| 1. Dissect and divide right pedicle |
| 1. Dissect and divide left pedicle |
| 1. Drop the bladder (fully dissected) |
| 1. Perform right-sided lymph node dissection (level 3) |
| 1. Divide the urethra |
| 1. Ligate the DVC with suture |
| 1. Place bladder into retrieval bag for extraction^a^ |
| 1. Oversew the DVC and vessels for haemostasis |
| 1. Spatulation of the ureters (using suture) |
| 1. Prepare bowel for stapled dissection |
| 1. Staple the bowel to form new bladder pouch |
| 1. Begin anastomosis of the ureters to the bladder pouch |
| 1. Insert ureteric stents |
| 1. Complete anastomosis of ureters to bladder pouch |

^a^Step not applicable since it is not necessary to remove the bladder from the cadaver. BSU: bedside unit; DVC: dorsal venous complex; VAS: vas deferens.
